# Rhinovirus infection presenting with symptomatic hyponatremia—Atypical presentation of a common infection

**DOI:** 10.1002/ccr3.8773

**Published:** 2024-04-22

**Authors:** Koutaibah Obaid, Mohamad Talal Basrak, Bisher Sawaf, Hiba Habib, Mohammed Alkhatib, Anas A. Ashour, Mhd Baraa Habib

**Affiliations:** ^1^ Internal Medicine Department Hamad Medical Corporation Doha Qatar; ^2^ Emergency Department Hamad Medical Corporation Doha Qatar; ^3^ Internal Medicine Department Al‐Mowasat Hospital Damascus Syria

**Keywords:** atypical presentation, hyponatremia, infection, rhinovirus, SIADH

## Abstract

**Key Clinical Message:**

Rhinovirus infection has the potential to exhibit unconventional symptoms like symptomatic hyponatremia. Health care professionals should remain vigilant about this potential complication, especially in cases with uncommon presentations. Timely identification and effective management of hyponatremia can mitigate potential complications and enhance patient prognosis.

**Abstract:**

The syndrome of inappropriate secretion of antidiuretic hormone (SIADH) is a prominent contributor to low sodium levels. Various factors can contribute to hyponatremia, affecting the diagnosis and treatment of the condition. Of note, some infections have been identified as potential causes of SIADH. Although rhinovirus infection has been linked to SIADH, it is usually associated with severe respiratory infections. Herein, we present a distinctive case where rhinovirus caused significant hyponatremia symptoms, even in the absence of typical respiratory symptoms or fever.

## 1 INTRODUCTION

Euvolemic hyponatremia, characterized by low plasma osmolality, elevated urine osmolality, increased natriuresis, hypouricemia, and the absence of other hyponatremic disorders, signifies the presence of the syndrome of inappropriate antidiuretic hormone secretion (SIADH).^1^ The primary cause of SIADH is the persistent release of antidiuretic hormone despite decreased serum osmolality. The origin of heightened antidiuretic hormone (ADH) secretion can be classified into pituitary and non‐pituitary sources.^2^ SIADH can be triggered by various medications, endocrine conditions, paraneoplastic syndromes, pneumonia, and central nervous system abnormalities.^1^ However, most frequently, SIADH is linked to an ongoing pathological process within the body.^2^ Additionally, several viral infections, including COVID‐19 and influenza, have been associated with SIADH.^3^, ^4^

Rhinoviruses are a prevalent cause of the common cold. While rhinovirus infections generally follow a self‐limiting course, certain situations can lead to complications such as severe respiratory symptoms and pneumonia.^5^ Like other viral pneumonias, cases of hyponatremia have been documented in conjunction with rhinovirus infections necessitating hospitalization due to severe pneumonia.^6^ Nevertheless, instances where hyponatremia symptoms present as the initial manifestations, without the typical signs of upper respiratory tract infection or pneumonia, are exceedingly rare.

In this case, we present the scenario of a middle‐aged patient who exhibited severe symptomatic hyponatremia as presenting symptoms for rhinovirus infection without overt respiratory symptoms or fever.

## 2 CASE REPORT

### 2.1 Presentation and investigations

**FIGURE 1:**
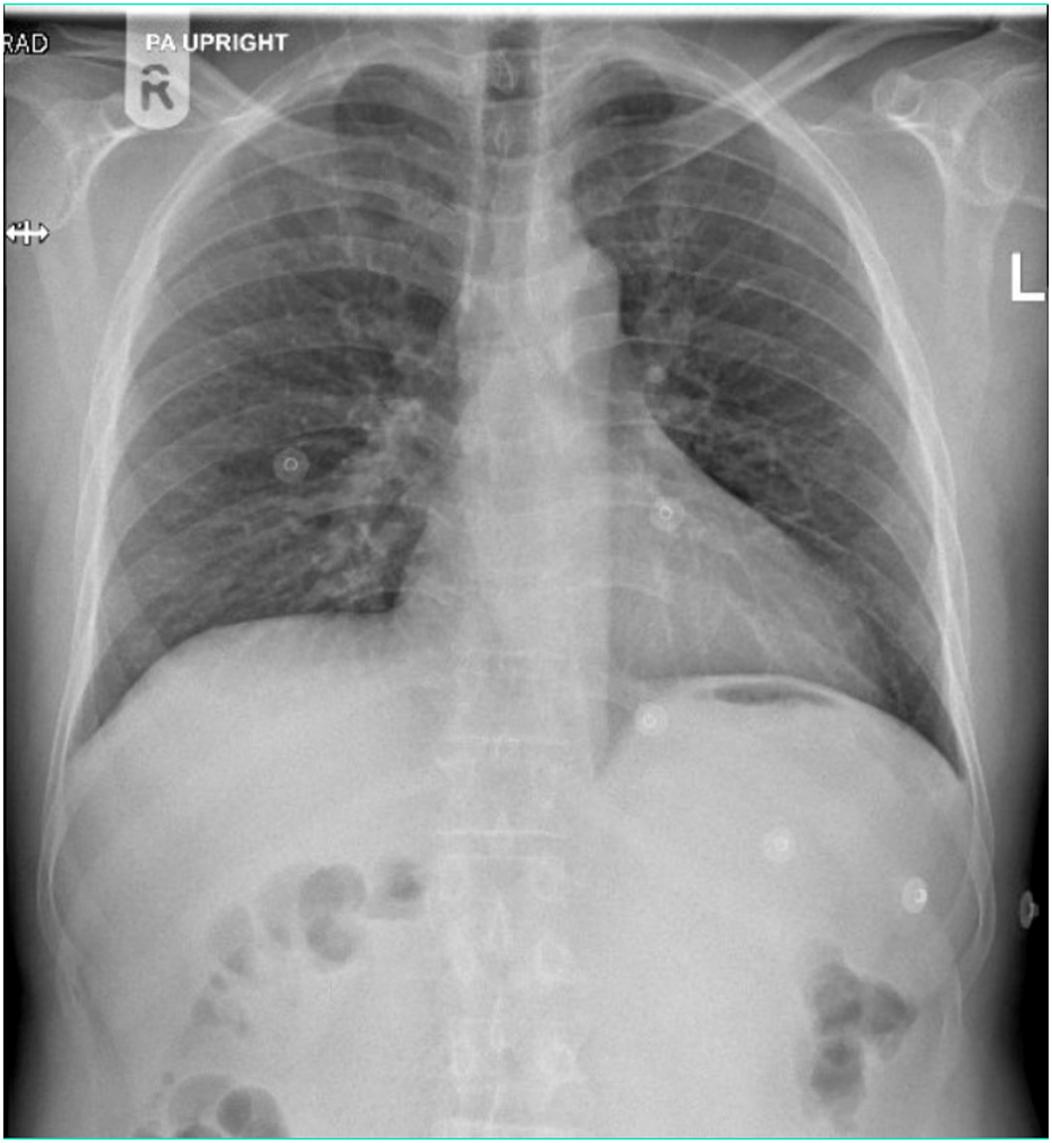
Chest x‐ray showing scattered patchy parenchymal infiltrates mostly in the right lower zone.

A 41‐year‐old man, without any preexisting chronic health conditions, arrived at the emergency department reporting a burning sensation behind the breastbone, discomfort in the upper abdomen, nausea, and dizziness persisting for 2 days. He had experienced two episodes of vomiting on the day he presented. He denied having a fever, cough, difficulty breathing, chest pain, sore throat, or a runny nose. Although he was not on any regular medications, he had recently been prescribed betahistine and domperidone for ongoing dizziness by a private clinic the day before, without experiencing any relief. Upon admission, vital signs showed a blood pressure of 123/83 mmHg, a heart rate of 78 beats per minute, a respiratory rate of 18, and an oxygen saturation of 99% on room air. The patient appeared to have a normal fluid balance including moist mucous membranes, normal skin turgor, and capillary refill time. During the chest examination, fine inspiratory crackles were detected in the lower right lung zone. Cardiac, gastrointestinal, and neurological exams were grossly unremarkable. Blood tests showed severe hyponatremia at a level of 114 meq/L, low chloride levels at 79 mmol/L, a mild elevation in leukocyte count at 10.9 × 10^3^/μL, with an absolute neutrophilic count of 7.3 × 10^3^/μL, a lymphocyte count of 2.4 × 10^3^/μL, and an eosinophil count of 0.16 × 10^3^/μL. Additionally, C‐reactive protein was slightly elevated at 11.2 mg/L. The rest of the blood counts and electrolytes were otherwise unremarkable (Table 1). A comprehensive workup for the hyponatremia causes revealed a serum osmolality of 238 mmol/kg, urine osmolality of 211 mmol/kg, and urine sodium concentration of 85 mmol/L (Table 2). Additionally, the patient exhibited normal levels of serum protein, glucose, thyroid function tests, and cortisol level. A chest x‐ray revealed scattered patchy infiltrates in the lung tissue (Figure 1). Nasopharyngeal swab testing was positive solely for rhinovirus, while other respiratory virus PCR tests yielded negative results (Table 3).

**TABLE 1:** General blood tests on presentation.

Detail	Value	Normal range
White blood count	10.9 × 10^3^/μL	4–10
Hemoglobin	14.6 gm/dL	13–17
Platelets	183 × 10^3^/μL	150–410
Calcium (adjusted)	2.21 mmol/L	2.2–2.6
Magnesium	0.79 mmol/L	0.7–1
C‐reactive protein	11.2 mg/L	0–5
HbA1C %	5.4%	< 5.7
pH, venous	7.344	7.32–7.42
Lipase	45 U/L	13–60
Urea	2.7 mmol/L	2.5–7.8
Creatinine	59 μmol/L	62–106
Sodium	114 mmol/L	133–146
Potassium	3.9 mmol/L	3.5–5.3
Chloride	79 mmol/L	95–108
Bicarbonate	22 mmol/L	22–29
Bilirubin, total	20 μmol/L	0–21
Albumin	43 gm/L	35–50

**TABLE 2:** Hyponatremia workup.

Detail	Value w/units	Normal range
Uric acid	165 μmol/L	200–430
Urine osmolality	211 mmol/kg	150–1150
Serum osmolality	238 mmol/kg	275–295
Urine sodium	85 mmol/L	

**TABLE 3:** Respiratory viral panel results.

Virus name	Result
Adenovirus	Negative
Influenza virus A (PH1N1, H1, H3)	Negative
Influenza virus B	Negative
Coronavirus (NL63, 229E, OC43, HKU)	Negative
Parainfluenza virus (1–4)	Negative
Human metapneumo virus	Negative
Boca virus	Negative
Mycoplasma pneumoniae	Negative
Respiratory syncytial virus	Negative
Human rhino virus	Positive
Bordetella pertussis	Negative
Legionella pneumophila	Negative
MERS coronavirus	Negative
COVID‐19 PCR	Negative

### 2.2 Treatment

**FIGURE 2:**
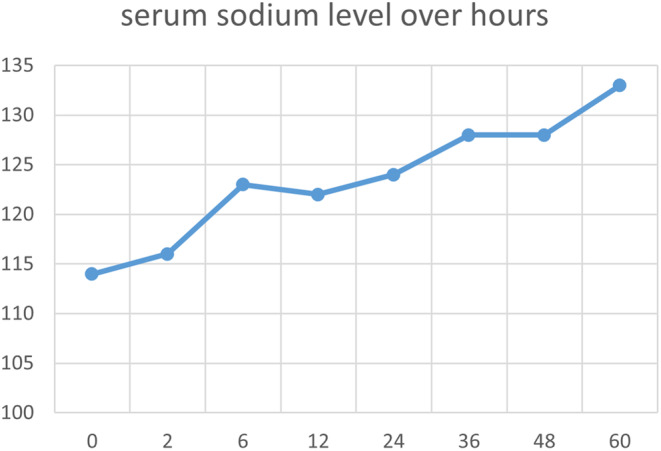
Sodium level trend.

To address the hyponatremia, the patient initially received 1 L of normal saline within an hour with a repeat sodium level showing 116 meq/L. Subsequently, he was placed on 250 mL of 2% sodium chloride, which elevated his sodium to 122 meq/L after 6 h. To prevent the complications associated with rapid correction of hyponatremia, such as osmotic demyelination syndrome, the patient was administered 500 mL of 5% dextrose in water over 3.3 h, after which his sodium level stabilized at 122 meq/L. As the diagnostic workup for hyponatremia was consistent with syndrome of inappropriate antidiuretic hormone secretion (SIADH), the patient was placed on a fluid restriction regimen of up to one liter per day. The patient's serum sodium levels were closely monitored over the subsequent hours. Figure 2 demonstrates the progression of correction.

### 2.3 Outcome and follow‐up

As the serum sodium level was corrected, the patient's symptoms notably improved. He did not develop any respiratory symptoms throughout the hospital stay. He was eventually discharged from the hospital with no ongoing symptoms and a sodium level of 133 meq/L. A follow‐up appointment with his primary care physician was scheduled 2 weeks later, at which point his sodium level had risen to 136 meq/L.

## 3 DISCUSSION

This case report sheds light on a rare yet potentially serious consequence of rhinovirus infection; the emergence of hyponatremia. Hyponatremia, a prevalent electrolyte imbalance, can stem from various factors such as medications, adrenal insufficiency, and infections.^7^ However, to date, no instances of hyponatremia induced by mild rhinovirus infection have been documented. Moreover, this is the inaugural case detailing an occurrence of isolated hyponatremia symptoms as a result of a rhinovirus infection.

The patient initially complained of dizziness, nausea, and vomiting. Although he received symptomatic treatment, his condition did not improve, leading to his hospitalization for comprehensive evaluation. Notably, he had no history of substance use or fluid intake irregularities. Laboratory tests unveiled severe hyponatremia, reflected by a serum sodium level of 114 mmol/L. Additional assessments of liver function, thyroid function, and serum creatinine remained within normal parameters. The diagnosis of SIADH was reached based on criteria including hypoosmolar hyponatremia, urine osmolality exceeding 100 mOsm/kg, urine sodium concentration surpassing 40 mEq/L, euvolemic state, and unremarkable renal and thyroid function.^8^

Although the patient lacked preceding viral illness symptoms, chest examination detected inspiratory crackles, and a chest x‐ray unveiled patchy infiltrates. Consequently, rhinovirus testing via PCR confirmed the diagnosis of rhinovirus infection. Despite the absence of respiratory or other associated symptoms, more sinister causes like adrenal insufficiency, hidden malignancies, or tumors were not considered. This decision was made based on the facts that the patient was middle‐aged, the presentation was acute, the hyponatremia improved with symptomatic treatment, and the normal sodium level was maintained post‐discharge.

While hyponatremia has been linked to viral infections such as influenza,^3^, ^10^ the precise mechanisms for rhinovirus‐induced hyponatremia remain less understood. An inferred mechanism involves the induced inflammatory response often seen in viral infections.^9^ Inflammation has been correlated with elevated interleukin (IL)‐6 levels, known to stimulate hypothalamic magnocellular neurons to release ADH.^9^ Additionally, secretion of other inflammatory cytokines like IL‐1 beta, IL‐2, and tumor necrosis factor alpha has been observed to enhance ADH release by stimulating parvocellular and magnocellular arginine vasopressin neurons.^10^

The unique aspect of this case report is that the patient solely exhibited symptoms of hyponatremia, without typical respiratory symptoms like cough or shortness of breath associated with rhinovirus infections. Furthermore, no evidence suggested concurrent bacterial infection, which usually elevates inflammatory markers and cytokines that influence ADH release.^11^ Thus, the patient's condition is a standout example of rhinovirus infection manifesting exclusively as hyponatremia symptoms, an exceedingly rare occurrence in the literature. This emphasizes the need to consider rhinovirus as a potential cause of hyponatremia, especially when other identifiable causes are absent.

In closing, health care practitioners should recognize the potential for severe complications linked to rhinovirus infections, including the development of hyponatremia. Patients displaying signs of hyponatremia warrant a thorough clinical assessment and relevant laboratory testing to pinpoint the underlying cause. Future research is crucial to unravel the pathophysiological mechanisms responsible for rhinovirus‐induced hyponatremia and to establish effective strategies for its prevention and management.

## 4 CONCLUSION

Exposure to rhinovirus can manifest in atypical symptoms like symptomatic hyponatremia. It's crucial for healthcare providers to stay alert to this possibility, particularly when faced with unusual symptoms. Prompt recognition and proper treatment of hyponatremia can help reduce the risk of complications and improve the outlook for patients.

## AUTHOR CONTRIBUTIONS

**Koutaibah Obaid:** Conceptualization; investigation; methodology; writing – original draft; writing – review and editing. **Mohamad Talal Basrak:** Conceptualization; methodology; writing – original draft; writing – review and editing. **Bisher Sawaf:** Data curation; investigation; writing – original draft; writing – review and editing. **Hiba Habib:** Resources; writing – original draft; writing – review and editing. **Mohammed Alkhatib:** Visualization; writing – original draft; writing – review and editing. **Anas A. Ashour:** Visualization; writing – original draft; writing – review and editing. **Mhd Baraa Habib:** Project administration; supervision; writing – review and editing.

## FUNDING INFORMATION

This research did not receive any specific grant from funding agencies in the public, commercial, or not‐for‐profit sectors.

## CONFLICT OF INTEREST STATEMENT

The authors have no conflict of interest to declare.

## ETHIC STATEMENT

The case was approved for publication by Hamad Medical Corporation IRB.

## CONSENT

Written informed consent was obtained from the patient to publish this report in accordance with the journal's patient consent policy.

## Data Availability

Data sharing is not applicable to this article as no datasets were generated or analyzed during the current study.
